# Serum Antibody Binding and Cytotoxicity to Pig Cells in Chinese Subjects: Relevance to Clinical Renal Xenotransplantation

**DOI:** 10.3389/fimmu.2022.844632

**Published:** 2022-03-10

**Authors:** Tao Li, Hao Feng, Jiaxiang Du, Qiangbing Xia, David K. C. Cooper, Hongtao Jiang, Songzhe He, Dengke Pan, Gang Chen, Yi Wang

**Affiliations:** ^1^ Department of Organ Transplantation, The Second Affiliated Hospital of Hainan Medical University, The Transplantation Institute of Hainan Medical University, Haikou, China; ^2^ Institute of Organ Transplantation, Tongji Hospital, Tongji Medical College, Huazhong University of Science and Technology, Wuhan, China; ^3^ Key Laboratory of Organ Transplantation, Ministry of Education and National Health Commission (NHC), Chinese Academy of Medical Sciences, Wuhan, China; ^4^ Genetic Engineering Department, Chengdu Clonorgan Biotechnology Co., Ltd., Chengdu, China; ^5^ Center for Transplantation Sciences, Department of Surgery, Massachusetts General Hospital/Harvard Medical School, Boston, MA, United States; ^6^ Clinical Immunology Translational Medicine Key Laboratory of Sichuan Province, Sichuan Academy of Medical Sciences & Sichuan Provincial People’s Hospital, Chengdu, China; ^7^ Department of Urology, Second Affiliated Hospital of University of South China, Hengyang, China

**Keywords:** brain-dead organ donors, complement-mediated cytotoxicity, end-stage renal disease, kidney, pig, xenotransplantation

## Abstract

Kidney xenotransplantation is expected to contribute to resolving the shortage of kidneys from deceased human donors. Although progress in experimental life-supporting pig renal xenotransplantation has been encouraging, there are still issues to be considered before a clinical trial can be initiated. We attempted to clarify some of these by an *in vitro* study. Blood was drawn from healthy volunteers (Volunteers, n=20), patients with end-stage renal disease (ESRD, n=20) pre-operation (Pre), and on Day 1 (POD 1) and Day 14 (POD 14) after renal allotransplantation, brain-dead organ donors (DBD, n=20), and renal allotransplant recipients who were currently experiencing T cell-mediated rejection (Allo-TCMR, n=20). Serum IgM/IgG binding to, and complement-dependent cytotoxicity (CDC) of, PBMCs and RBCs from (a) wild-type (WT), (b) α1,3-galactosyltransferase gene-knockout (GTKO), (c) GTKO/beta-1,4-N-acety1 galactosaminyltransferase 2-knockout (GTKO/β4GalNT2KO), (d) GTKO/cytidine monophosphate-N-acetylneuraminic acid hydroxylase-knockout (GTKO/CMAHKO), and (e) GTKO/β4GalNT2KO/CMAHKO/hCD55 (TKO/hCD55) pigs were measured by flow cytometry. We obtained the following results: (i) Serum IgM/IgG binding and CDC in Volunteers were significantly greater to WT, GTKO, and GTKO/β4GalNT2KO PBMCs or RBCs than to GTKO/CMAHKO and TKO/hCD55 cells; (ii) ESRD, DBD, and Allo-TCMR serum antibody binding and CDC to WT pig PBMCs were significantly greater than to GTKO, GTKO/β4GalNT2KO, GTKO/CMAHKO, and TKO/hCD55 cells; (iii) antibody binding to GTKO/CMAHKO pig cells was significantly lower in hemodialysis than peritoneal dialysis patients. (iv) Two of twenty allotransplantation recipients’ serum IgG binding to GTKO pig PBMCs increased on POD14 compared with Pre, but IgG binding to GTKO pig RBCs did not; (v) In all sera, the lowest antibody binding and CDC were to GTKO/CMAHKO and TKO/CD55 pig cells. We conclude (i) CMAHKO in the pig may be critical to the success of clinical pig kidney xenotransplantation, and may be the most important after GTKO, at least in Chinese patients; (ii) subjects with ESRD, or who are immunosuppressed after kidney allotransplantation, and DBD, have lower levels of antibody binding and CDC to genetically-engineered pig cells than do volunteers; (iii) TKO pigs with selected human ‘protective’ transgenes, e.g., CD55, are likely to prove to be the optimal sources of kidneys for clinical xenotransplantation.

## Introduction

There is a critical shortage of deceased human donor organs for transplantation in patients with end-stage renal disease (ESRD) (1). Genetically-engineered pigs are a potential alternative source of kidneys for these patients. Pig-to-nonhuman primate kidney transplantation Is now associated with encouraging results with recipient and graft survival extending to >1 year in several cases (2–4). However, there are still some major issues that must be resolved before a clinical trial can be initiated, e.g., (i) what genetically-modified pigs should be the sources of kidneys for clinical renal xenotransplantation; and (ii) whether a new xenotransplantation model needs to be identified because of differences in antibody binding and complement-dependent cytotoxicity (CDC) to pig cells between humans and Old World monkeys (OWMs) (5).

Pigs that do not express Gal or Sda (GTKO/β4GalNT2KO pigs), with or without added human transgenes, may be the optimal source of organs for OWMs (**Table 1**), whereas pigs in which expression of all 3 known carbohydrate xenoantigens has been deleted [triple-knockout (TKO) pigs], with or without added human transgenes, are likely to be optimal for human recipients (5–8). Humans have low (or no) antibody levels and CDC to cells from TKO pigs.

**Table 1:** Sources of human sera and types of pig cells used in these studies.

*Human sera tested (and abbreviations used)*
1. Healthy volunteers (Volunteers)
2. Patients with end-stage renal disease (ESRD) pre-kidney allotransplantation (Pre), and post-kidney allotransplantation on day 1 (POD1) and on day 14 (POD14)
3. Brain-dead organ donors (DBD)
4. Patients with kidney allografts that were currently experiencing acute T cell-mediated rejection (Allo-TCMR).
*Pig cells (PBMCs and RBCs) tested (and abbreviations used)*
1. Wild-type (WT, i.e., genetically-*un*modified)
2. α1,3-galactosyltransferase gene-knockout (GTKO)
3. GTKO/β-1,4N-acetylgalactosaminyltransferase gene-knockout (GTKO/β4GalNT2KO)
4. GTKO/cytidine monophosphate-N-acetylneuraminic acid hydroxylase gene-knockout (GTKO/CMAHKO).
5. Triple knockout (i.e., GTKO/β4GalNT2KO/CMAHKO) + transgenic expression of the human complement-regulatory protein, CD55 (TKO/hCD55).

Whether or not swine leukocyte antigen (SLA) expression needs to be deleted remains uncertain. SLA is the homolog of human leukocyte antigen (HLA), a protein complex expressed on human tissue capable of stimulating the development of new antibodies in allotransplantation. Some *in vitro* studies have indicated that HLA-sensitized patients will not be at greater risk of rejecting a pig organ than HLA-non-sensitized patients (9–14), but other studies indicate that HLA-sensitized patients have a greater risk of rejecting a pig organ (15–17), and so it would be prudent not to select HLA-sensitized patients for the first clinical trials of pig kidney transplantation (18).

Subjects with brain-death (DBD subjects) are a frequent source of organs for transplantation, and transplantation of a pig organ into a brain-dead human *recipient* has recently been carried out (19). However, brain death is associated with dysfunction of the cardiovascular, pulmonary, endocrine, thermoregulation, renal, hematologic and inflammatory systems (20–23). If DBD subjects are used as *recipients* in preclinical models of pig renal xenotransplantation, there is concern that these pathophysiological consequences may affect the xenograft, e.g., by activation of T and B lymphocytes, release of cytokines, etc. (19).

The aims of the present study were (i) to measure serum anti-pig antibodies in healthy human volunteers, patients with ESRD pre- and post-renal allotransplantation, DBD subjects, and patients with renal allografts who were currently experiencing acute T cell-mediated rejection (Allo-TCMR), and (ii) to provide further data to help select pigs with the optimal genotype for clinical renal xenotransplantation.

## Materials and Methods

### Human Sera

Blood was drawn from (i) healthy volunteers (Volunteers, n=20; ABO blood types A n=6; B n=6; AB n=3; O n=5), (ii) patients with ESRD (n=20), pre-renal transplantation (Pre) and on Day 1 (POD 1) and Day 14 (POD 14) after renal allotransplantation, (iii) brain-dead organ donors (DBD, n=20) and (iv) patients with renal allografts who were currently experiencing episodes of acute T cell-mediated rejection (Allo-TCMR, n=20) (**Table 1**). Sera were obtained from de-identified remnant/discarded clinical laboratory samples.

Sera from Volunteers were obtained from the Second Affiliated Hospital of Hainan Medical University, and all experimental protocols were approved by the ethics committee of the Second Affiliated Hospital of Hainan Medical University. All procedures involving humans were performed in accordance with the relevant guidelines and regulations, and had no adverse effects on the subjects.

### Pigs

Blood was obtained from wild-type (WT, i.e., genetically-*un*modified) pigs (n=4) and from different genetically-modified pigs (n=4) (**Table 1**) (Chengdu Clonorgan Biotechnology, Chengdu, Sichuan, China).

### Detection of Expression of Xenoantigens on Selected Pig Cells by Flow Cytometry

Pig RBCs and PBMCs were stained for expression of Gal (by isolectin BSI-B4), Sda (Dolichos biflorus agglutinin,DBA), Neu5Gc (chicken anti-Neu5Gc mAb), and SLA (anti-human β2-microglobulin antibody, β2M).

### Binding of Human Serum IgM and IgG to pRBCs and pPBMCs by Flow Cytometry

Binding of human antibodies to pig cells was measured by flow cytometry using the relative geometric mean (rGM), as previously described (11). Briefly, pRBCs were separated from whole blood, washed x3 with phosphate-buffered saline (PBS), and centrifuged at 700g for 5min at 4°C. The washed RBCs were suspended in fluorescence-activated cell sorting (FACS) buffer (PBS containing 1% bovine serum albumin). pPBMCs were isolated using Ficoll (HaoYang, Tianjin, China) and suspended in FACS buffer for IgM/IgG binding assays. The isolated pRBCs (5x10^5^/tube) and pPBMCs (5x10^5^/tube) were incubated with heat-inactivated human serum at 4°C for 30min, respectively, and the final serum concentration was 20%. After incubation, cells were washed with PBS to remove unbound antibodies and were blocked with 10% goat serum for 15min at 4°C. After further washing with PBS, anti-human IgM or anti-human IgG (Jackson ImmunoResearch Laboratories, West Grove, PA, USA) (IgG: concentration 1:1000 for pRBCs and pPBMCs; IgM: concentration 1:1600 for pRBCs and pPBMCs) was added, and the cells were incubated for 30min at 4°C. After washing with PBS, 100μL PBS buffer was added. Flow cytometry was carried out using BD FACSCelesta (Becton Dickinson, San Jose, CA, USA).

### Human Serum CDC of Pig PBMCs by Flow Cytometry

Briefly, PBMCs (5×10^5^ cells in 250µL FACS buffer) were incubated with 50µL heat-inactivated human serum at 4°C for 1h. After washing with PBS, FACS buffer (200µL) and rabbit complement (50µL, Cedarlane, Hornby, CA, USA) were added (final concentration 20%), and incubation was carried out at 37°C for 30min. After washing with PBS, the cells were incubated in the dark at 4°C for 15min with propidium iodide, and finally 200µL FACS buffer was added. Flow cytometry was carried out using BD FACSCelesta.

Cytotoxicity was calculated, as follows (11):


% cytotoxicity=([A−C]/[B−C])×100

where A represented the percentage of dead cells, B was the maximal percentage of dead cells (PBMCs fixed with 70% ethanol), and C was the minimal percentage of dead cells (PBMCs incubated with medium only).

### Statistical Analysis

Significance of the difference between two groups was determined by student t-test or Wilcoxon test. Continuous variables were expressed as mean ± SD. Comparisons among multiple groups were performed using a One-way ANOVA test (Tukey test) or nonparametric test (Dunn’s test). A p value of <0.05 was considered statistically significant. All statistical analyses were performed using social sciences software GraphPad Prism 8 (GraphPad Software, San Diego, CA, USA).

## Results

### Expression of Gal, Sda, Neu5Gc, and SLA on Pig PBMCs and/or RBCs

#### PBMCs

**Figure 1:**
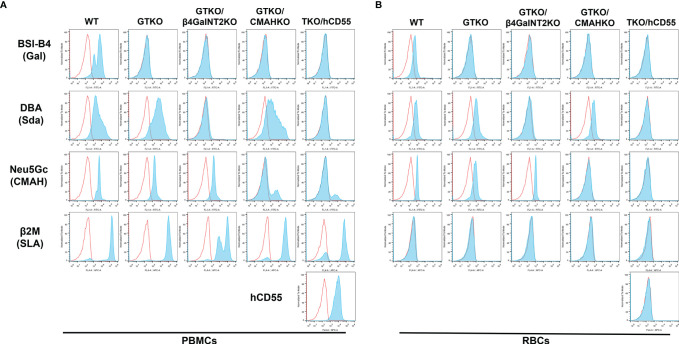
Expression of Gal, Sda, Neu5Gc, SLA, and hCD55 on WT, GTKO, GTKO/β4GalNT2KO, GTKO/CMAHKO and TKO/hCD55 pig PBMCs and RBCs by flow cytometry. **(A)** PBMCs and **(B)** RBCs from WT pigs expressed Gal, Sda, and Neu5Gc. GTKO expressed Sda and Neu5Gc. GTKO/β4GalNT2KO expressed Neu5Gc. GTKO/CMAHKO expressed Sda. TKO/hCD55 PBMCs (but not RBCs) expressed hCD55. All PBMCs expressed SLA, but RBCs did not express SLA.

PBMCs from WT pigs expressed Gal, Sda, Neu5Gc, and SLA (**Figure 1A**). As anticipated, PBMCs from a GTKO pig did not express the Gal antigen, but expressed Sda and Neu5Gc. Those from a GTKO/β4GalNT2KO pig did not express Gal or Sda, but expressed Neu5Gc. Those from a GTKO/CMAHKO pig did not express Gal or Neu5Gc, but expressed Sda. And those from a TKO/hCD55 pig did not express any of the 3 carbohydrate xenoantigens, but expressed hCD55. Neu5Gc that was still expressed a small amount in GKTO/CMAHKO and TKO/hCD55 pig PBMCs was a false positive which was caused by Rabbit anti-Chicken IgY/Alexa Fluor 555 antibody (**Supplemental Material**).

There was a positive expression The false positive expression of Neu5Gc on GTKO/CMAHKO and TKO/hCD55 pig PBMCs were caused by Rabbit anti-Chicken IgY/Alexa Fluor 555 antibody (**Supplemental Material**).

#### RBCs

Expression of xenoantigens on RBCs from all of the above pigs followed the same pattern as that to PBMCs except that they did not express SLA or hCD55 (**Figure 1B**).

### Effect of Different Human Sera on IgM and IgG Binding and CDC to WT and Various Genetically-Modified Pig PBMCs by Flow Cytometry

The aim was to compare the binding of different human sera to various pig PBMCs.

#### IgM Binding

**Figure 2:**
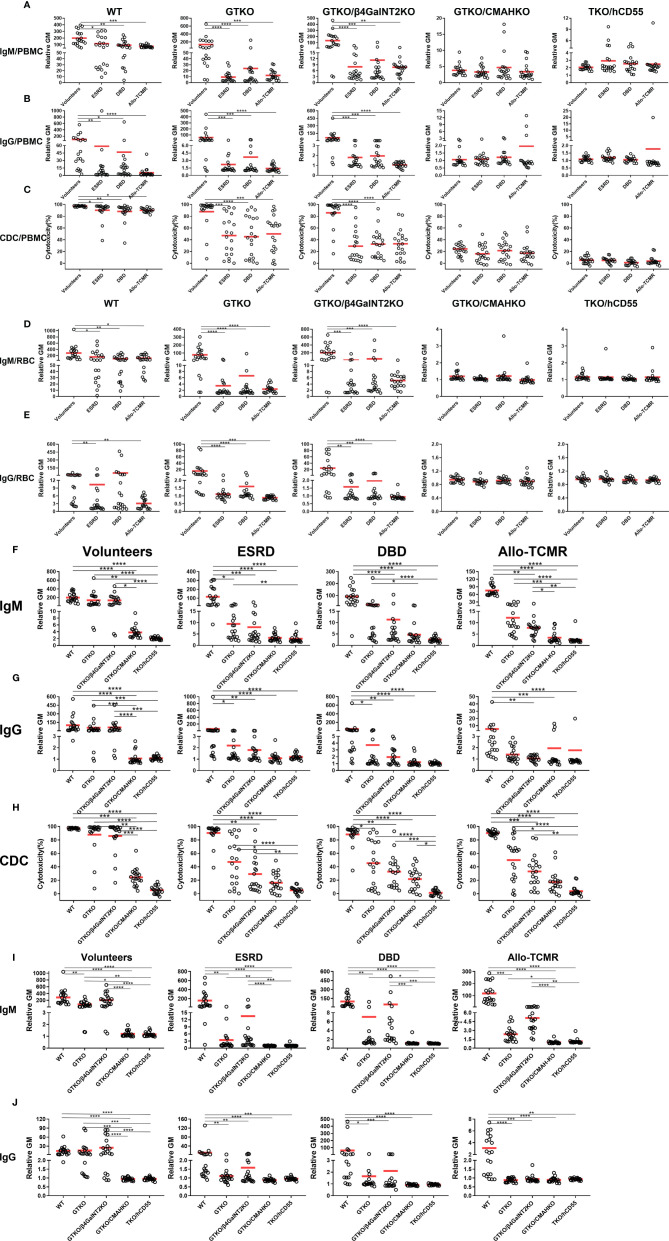
Effect of different human sera on IgM and IgG binding and CDC to various pig PBMCs and RBCs and effect of genetic-engineering of pig PBMCs and RBCs on human IgM and IgG binding and CDC by flow cytometry. Comparison of mean **(A)** IgM/**(B)** IgG binding and **(C)** CDC of sera from Volunteers, ESRD, DBD and Allo-TCMR to WT, GTKO, GTKO/β4GalNT2KO, GTKO/CMAHKO, and TKO/hCD55 pig PBMCs. Mean of **(D)** IgM and **(E)** IgG comparing binding of sera from Volunteers, ESRD, DBD, and Allo-TCMR to WT, GTKO, GTKO/β4GalNT2KO, GTKO/CMAHKO, and TKO/hCD55 pig RBCs. (*p <0.05, **p<0.01, ***p<0.005, ****p<0.001). Volunteers had very low levels of IgM/IgG binding and CDC to GTKO/CMAHKO and TKO/hCD55 pig PBMCs **(F-H)** or RBCs **(I, J)**. IgM and IgG binding and CDC to GTKO, GTKO/β4GalNT2KO, GTKO/CMAHKO, and TKO/hCD55 PBMCs **(F–H)** or RBCs **(I, J)** were low in sera from ESRD, DBD, and Allo-TCMR. (*p<0.05, **p<0.01, ***p<0.005, ****p<0.001).

Mean IgM binding to WT, GTKO and GTKO/β4GalNT2KO PBMCs was significantly greater in Volunteers than in the other three sera. Mean IgM binding to GTKO/CMAHKO and TKO/hCD55 PBMCs was minimal and not significantly different between all four groups of sera (Volunteers, ESRD, DBD, and Allo-TCMR) (**Figure 2A**).

#### IgG Binding

Mean IgG binding to WT, GTKO and GTKO/β4GalNT2KO PBMCs was significantly greater in Volunteers than in the other three sera. No sera showed more than minimal binding to GTKO/CMAHKO and TKO/CD55 PBMCs (**Figure 2B**).

#### CDC

Mean serum CDC to GTKO/CMAHKO and TKO/hCD55 pig PBMCs was not significantly different between Volunteers, ESRD, DBD, and Allo-TCMR (**Figure 2C**). However, mean CDC to WT, GTKO and GTKO/β4GalNT2KO pig PBMCs was significantly greater in Volunteers than in ESRD, DBD, and Allo-TCMR. CDC of GTKO/CMAHKO PBMCS was low or negative in all sera, and almost no sera caused any killing of TKO/CD55 PBMCs.

### Effect of Different Human Sera on IgM and IgG Binding to WT and Various Genetically-Modified Pig RBCs by Flow Cytometry

The aim was to compare the binding of different human sera to various pig RBCs.

#### IgM Binding

Mean IgM binding to WT, GTKO and GTKO/β4GalNT2KO RBCs was significantly greater in Volunteers compared with ESRD, DBD, and Allo-TCMR. Mean IgM binding to GTKO/CMAHKO and TKO/hCD55 RBCs was minimal in all sera and was not significantly different between all four groups of sera (**Figure 2D**).

#### IgG Binding

Mean IgG binding to WT was significantly greater in Volunteers compared with ESRD an Allo-TCMR. Mean IgG binding to GTKO and GTKO/β4GalNT2KO RBCs was significantly greater in Volunteers than in ESRD, DBD, and Allo-TCMR. Mean IgG binding was absent or minimal to GTKO/CMAHKO and TKO/CD55 RBCs (**Figure 2E**).

### The Effect of Genetic-Engineering of Pig PBMCs on Human IgM and IgG Binding and CDC by Flow Cytometry

The data presented in relation to differences in binding of various human sera to pig cells (**Figures 2A**–**E**) were re-presented to more clearly illustrate the effect of different pig genotypes (**Figures 2F**–**J**).

#### IgM Binding

Mean IgM binding in serum from Volunteers to WT, GTKO, and GTKO/β4GalNT2KO PBMCs was significantly greater than to GTKO/CMAHKO and TKO/hCD55 PBMCs. Mean IgM binding in serum from ESRD to WT PBMCs was significantly greater than to GTKO, GTKO/β4GalNT2KO, GTKO/CMAHKO and TKO/hCD55 PBMCs, and mean IgM binding to GTKO PBMCs was significantly greater than to TKO/hCD55 PBMCs. Mean IgM binding in serum from DBD and Allo-TCMR to WT PBMCs was significantly greater than to GTKO/β4GalNT2KO, GTKO/CMAHKO and TKO/hCD55 PBMCs, and the mean IgM binding to GTKO PBMCs was significantly greater than to GTKO/CMAHKO and TKO/hCD55 PBMCs, what’s more, Mean IgM binding in serum from Allo-TCMR to GTKO/β4GalNT2KO PBMCs was significantly greater than to GTKO/CMAHKO and TKO/hCD55 PBMCs (**Figure 2F**).

In healthy human sera (Volunteers), mean IgM binding was reduced by approximately 20% by GTKO (compared to WT), but by approximately 80% by CMAHKO (WT vs GTKO: 199 vs 138, p=ns; WT vs CMAHKO: 199 vs 4, p<0.001) (**Figure 2F**).

#### IgG Binding

**Figure 3:**
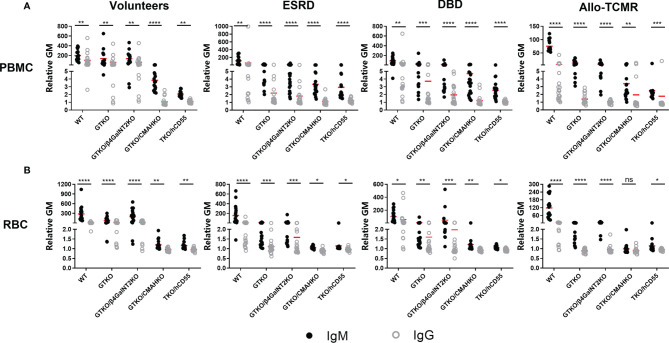
Comparison of serum IgM and IgG binding to PBMCs and RBCs. All human subjects had lower levels of IgG binding (compared to IgM) to WT, GTKO, GTKO/β4GalNT2KO, GTKO/CMAHKO, and TKO/hCD55 PBMCs **(A)** and RBCS **(B)**. (*p<0.05, **p<0.01, ***p<0.005, ****p<0.001).

Mean IgG binding was almost lower than mean IgM binding in all sera (**Figure 3A**). Mean IgG binding in Volunteers to WT, GTKO and GTKO/β4GalNT2KO PBMCs was significantly greater than to GTKO/CMAHKO and TKO/hCD55 PBMCs. Mean IgG binding in ESRD and DBD to WT PBMCs was significantly greater than to GTKO, GTKO/β4GalNT2KO, GTKO/CMAHKO and TKO/hCD55 PBMCs. Mean IgG binding in serum from Allo-TCMR to WT PBMCs was significantly greater than to GTKO/β4GalNT2KO, GTKO/CMAHKO and TKO/hCD55 PBMCs (**Figure 2G**). In all sera, binding to GTKO/CMAHKO and TKO/CD55 PBMCs was minimal or absent.

In healthy human sera (Volunteers), mean IgG binding was reduced by approximately 5% by GTKO (compared to WT) but by approximately 90% by CMAHKO (WT vs GTKO: 100 vs 58, p=ns; WT vs CMAHKO: 100 vs 1, p<0.01)

#### CDC

Although there were many variations in CDC depending on the serum and the source of PBMCs (**Figure 2H**), the most obvious finding was that CDC to GTKO/CMAHKO and, particularly, TKO/CD55 PBMCs was generally low or absent.

In healthy human sera (Volunteers), mean CDC was reduced by approximately 10% by GTKO (compared to WT) but by approximately 80% by CMAHKO (WT vs GTKO: 97 vs 88, p=ns; WT vs CMAHKO: 97 vs 25, p<0.001).

### The Effect of Genetic-Engineering of Pig RBCs on Human IgM and IgG Binding by Flow Cytometry

#### IgM Binding

Mean serum IgM binding followed the same pattern as for IgM binding to PBMCs, with minimal binding to GTKO/CMAHKO and TKO/CD55 RBCs in all sera (**Figure 2I**).

#### IgG Binding

Mean serum IgG binding was lower in all sera than IgM binding (**Figure 3B**). Binding was greatest in the sera from Volunteers, but again was minimal to GTKO/CMAHKO and TKO/CD55 RBCs (**Figure 2J**).

### Comparison of IgM/IgG Antibody Binding and CDC to Pig PBMCs or RBCs Between Hemodialysis Patients and Peritoneal Dialysis Patients

Patients with ESRD were divided into 2 groups based on whether they were undergoing hemodialysis (n=15) or peritoneal dialysis (n=5).

#### IgM/IgG Binding

**Figure 4:**
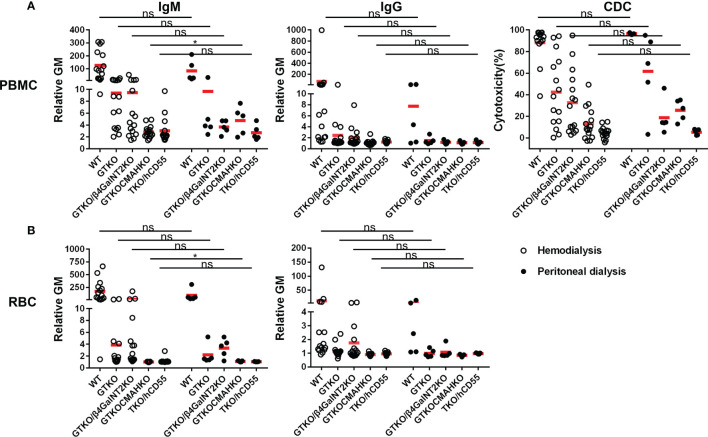
Comparison of serum IgM/IgG binding and CDC to PBMCs or RBCs from WT, GTKO, GTKO/β4GalNT2KO, GTKO/CMAHKO and TKO/hCD55 pig in patients receiving hemodialysis or peritoneal dialysis. **(A)** There were no differences in IgM/IgG binding or CDC to WT, GTKO, GTKO/β4GalNT2KO, and TKO/hCD55 PBMCs, but IgM binding to GTKO/CMAHKO to PBMCs was significantly lower in hemodialysis patients. **(B)** There were no differences in IgM/IgG binding to WT, GTKO, GTKO/β4GalNT2KO, GTKO/CMAHKO, and TKO/hCD55 RBCs, but IgM binding to GTKO/CMAHKO to RBCs was again significantly lower in hemodialysis patients. (*p<0.05; ns=not significant).

There were no significant differences in IgM/IgG binding to WT, GTKO, GTKO/β4GalNT2KO, and TKO/hCD55 PBMCs and RBCs between hemodialysis and peritoneal dialysis patients (**Figure 4**), but serum IgM binding to GTKO/CMAHKO PBMCs and RBCs was significantly lower in hemodialysis patients than in peritoneal dialysis patients.

#### CDC

There was no significant difference in CDC to any cell type between hemodialysis and peritoneal dialysis patients (**Figure 4**).

### Changes in the Levels of IgM and IgG Binding in Patients With ESRD Who Underwent Kidney Allotransplantation

**Figure 5:**
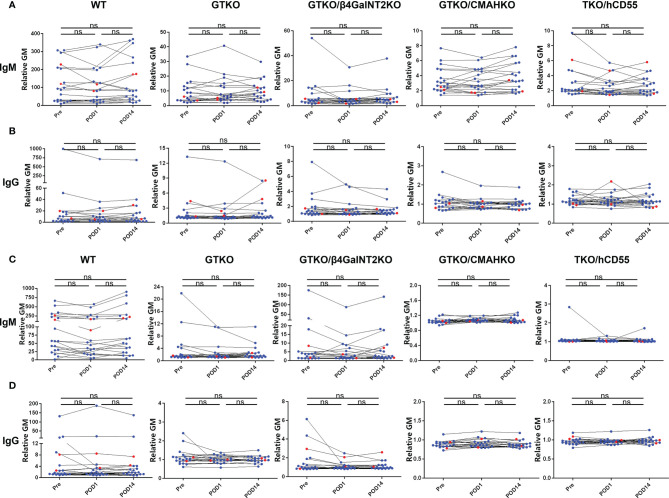
Changes in IgM and IgG binding to pig cells in patients with ESRD pre and early post-renal allotransplantation. Serum samples from renal allotransplant recipients were collected Pre, and on POD1 and POD14 to measure IgM **(A)** and IgG **(B)** binding to pig PBMCs or to pig RBCs **(C, D)**. In the majority of cases, there were no significant changes in binding between the time-intervals, although in some there was a transient reduction in IgM and IgG binding on POD1 (possibly associated with hemodilution or binding to the pig cells) that had recovered to Pre levels by POD14. In two sera, IgG binding to GTKO PBMCs increased on POD14 compared with Pre and POD1, but there was no change in binding to GTKO RBCs, nor to pig cells of other genotypes. (ns, not significant).

Serum samples from renal allotransplant recipients were collected pretransplant (Pre), and on POD1 and POD14, and the levels of anti-pig IgM and IgG antibodies were measured (**Figure 5**). In the majority of patients (n=18), there was no change in IgM/IgG binding to PBMCs or RBCs between Pre, POD 1, and POD 14, although in some patients anti-pig IgM/IgG decreased transiently on POD1, but recovered to Pre levels by POD14. (This may possibly be related to hemodilution by perioperative fluid infusion.) However, in 2 of the recipients (red dot), serum IgG binding to GTKO PBMCs increased by POD14, compared with Pre and POD1. There was no change in IgG binding to GTKO RBCs (that do not express SLA), nor in binding to PBMCs and RBCs from the other pig genotypes.

### Influence of ABO Blood Type of Healthy Human Volunteers on Serum IgM and IgG Binding and CDC to WT and Various Genetically-Modified Pig PBMCs or RBCs by Flow Cytometry

Healthy volunteers were divided into 4 groups based on ABO blood type.

#### IgM/IgG Binding

**Figure 6:**
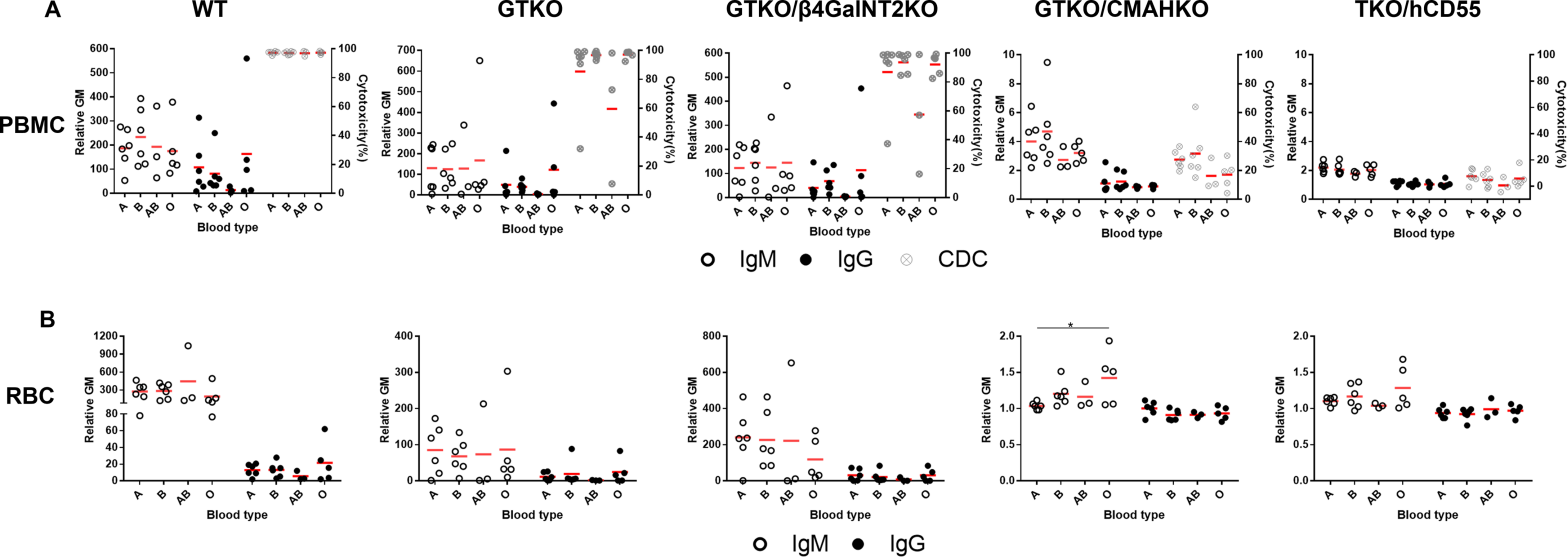
Comparison of IgM/IgG binding and CDC to PBMCs or RBCs from WT, GTKO, GTKO/β4GalNT2KO, GTKO/CMAHKO and TKO/hCD55 pigs in sera from healthy human volunteers of different ABO blood types. **(A)** There were no differences in IgM (left) or IgG (middle) binding or CDC (right) to WT, GTKO, GTKO/β4GalNT2KO, GTKO/CMAHKO, and TKO/hCD55 PBMCs in sera from humans of various ABO blood types. **(B)** There were no differences in IgM (left) or IgG (right) binding to WT, GTKO, GTKO/β4GalNT2KO, and TKO/hCD55 RBCs, but IgM binding to GTKO/CMAHKO RBCs was significantly lower in sera from A blood type subjects than in those of O blood type. (*p<0.05).

There were no significant differences in IgM/IgG binding to WT, GTKO, GTKO/β4GalNT2KO, and TKO/hCD55 PBMCs and RBCs in relation to ABO blood type, but IgM binding to GTKO/CMAHKO RBCs was significantly lower in sera from subjects with A blood type compared to those with O blood type (**Figure 6**).

#### CDC

There was no significant difference in serum CDC between subjects of the four blood types (**Figure 6**).

## Discussion

### Effect of CMAHKO

The first important observation in this study was that our data showed that healthy volunteer serum IgM binding to WT, GTKO, GTKO/β4GalNT2KO PBMCs was significantly greater than to CMAHKO and TKO/hCD55 PBMCs, strongly suggesting that CMAHKO will be important for clinical renal xenotransplantation, as others have also reported (24, 25). However, deletion of expression of Neu5Gc appeared to play a more important role in reducing human antibody binding to the pig cells than deletion of expression of Gal, which is in contrast to some previous studies in which GTKO had a much greater impact than CMAHKO on IgM binding (14, 26). The reduction in IgM and IgG binding to GTKO pig cells when compared to WT cells was less than reported in most previous studies (25), but, in contrast, the reduction in binding and CDC was significantly greater after deletion of expression of Neu5Gc. This is most likely explained by differences in the antibody profiles of the Chinese participants in this study compared with those of some other ethnic groups.

Neu5Gc is expressed in pigs, apes and OWMs, but not in humans (27–29), and therefore only humans develop anti-Neu5Gc antibodies (30). Gao and her colleagues drew attention to the fact that natural antibodies are largely associated with exposure to glycans expressed on flora in the gastrointestinal tract (26), as suggested by others previously (31). In humans, anti-Neu5Gc antibodies develop during the first 6 months of life, and reach adult levels by the end of the first year (32). Both anti-Neu5Gc IgM and IgG increase soon after the infant is exposed to cow’s milk and baby foods containing red meat, which express Neu5Gc (32).

All of the subjects from whom blood was drawn in the present study were Chinese and had been resident in China throughout most of their lives, whereas those in other reported studies were from a variety of ethnic and geographic backgrounds. This suggests that maybe in Chinese patients (or in patients who have been exposed to Chinese environmental factors, e.g., diet, for a prolonged period of time), an absence of expression of Neu5Gc in the pig organ may be as important, if not more important, than absence of expression of Gal.

We therefore suggest that the Chinese subjects included in the present study expressed different gastrointestinal flora (perhaps based on differences in diet), than other groups that have been studied in other geographic regions (26, 33), thus rendering Neu5Gc a more important stimulus to natural antibody production. However, of interest, no correlation between diet and anti-pig antibody levels was found in a previous study in Taipei (34). There may other ‘ethnic’ or ‘environmental’ differences in other population groups that have not yet been investigated.

There are increasing *in vitro* data indicating that TKO pig organs will prove to be a major advance over GTKO organs for transplantation into humans (5, 7, 35) which is consistent with our conclusions from the present study.

### Healthy Human Volunteers vs Other Groups

A second major observation made in this study was that (i) patients with ESRD, brain-dead donors, and immunosuppressed patients with kidney allografts generally had significantly lower levels of anti-pig antibodies than healthy human volunteers, except in regard to WT pig cells. The trends in CDC were similar to those of IgM and IgG. In addition, the ABO blood type of the donor of the serum appeared to play no part in influencing the results.

The clinical impact of ESRD on anti-pig immunity remains to some extent uncertain because immune dysfunction in ESRD includes both immunoactivation and immunosuppression. Heparin-induced antibodies (HIA) (36, 37) and anticardiolipin antibodies (IgG-ACA) (38) are elevated in ESRD and the complement system can be activated (39, 40). However, It is well-recognized that ESRD patients are to some extent immunocompromised (41, 42), e.g., reduced number of NK cells, reduced phagocytic activity of neutrophils  (43). Anti-pig antibodies are low in infants (44) and in patients with ESRD (14), the last of which observations is consistent with our present results.

Patients with kidney allografts are receiving chronic immunosuppressive therapy, and presumably this has prevented an increase in antibody levels even though T cell activation had taken place in the patients we studied. The explanation in brain-dead donors is not so obvious, but may be associated with the infusion of fluids to maintain an adequate hemodynamic state, resulting in hemodilution.

However, there was no difference in serum IgM/IgG binding to GTKO/CMAHKO and TKO/hCD55 PBMCs or RBCs in the 4 groups, indicating that the effect of deletion of Neu5Gc expression on the pig cell was sufficient to reduce antibody binding and CDC to negligible levels whatever the source of the serum. Expression of CD55 on TKO pig PBMCs further reduced CDC of the cells.

### Hemodialysis vs Peritoneal Dialysis

Our data showed that there was no significant difference in serum IgM/IgG binding to any of the pig cell types between hemodialysis and peritoneal dialysis patients, with the exception of lower IgM binding to GTKO/CMAHKO cells in patients on hemodialysis. This suggests that hemodialysis might remove anti-β4GalNT2 antibodies. However, it is unlikely that hemodialysis directly removes antibodies since standard hemodialysis filters typically have a cut-off size of between 10–20 kDa, whereas IgM/IgG molecules are >150 kDa, and are thus *not* removed by hemodialysis (45).

### Experimental Models of Pig Organ Xenotransplantation

As is well-known, the most widely used xenotransplantation model is the genetically-modified pig-to-OWM. A major difference between humans and OWMs is that OWMs express Neu5Gc on the vascular endothelium, whereas humans do not. OWMs, therefore, are far from ideal models for xenotransplantation, as there is markedly increased OWM serum antibody binding and CDC to TKO pig cells. Although New World monkeys have some advantage in this respect (5), their small size and the ineffectiveness of some immunosuppressive drugs in them negates their suitability as a surrogate recipient for pig organ transplantation (46–50). Is there an alternative recipient as a surrogate for living humans?

Could DBD subjects be used as *recipients* in preclinical studies of xenotransplantation? Our data indicate that their levels of anti-pig antibodies are significantly lower than healthy volunteers (possibly associated with hemodilution through the need for fluid administration to maintain hemodynamic stability), and comparable to those in patients with ESRD. However, activation of innate immunity and inflammation can occur in brain-dead subjects (21). This observation, and because of their hemodynamic instability that may limit follow-up to days rather than months, reduces their suitability as potential surrogates for living recipients (51, 52).

Our observation that 2 of 20 patients undergoing kidney allotransplantation developed increased anti-pig antibody binding on POD14 may suggest that, at least in some patients, there may be cross-reactivity between anti-HLA antibodies and SLA epitopes, but the data are too few to draw any definite conclusions. (Unfortunately, anti-HLA antibodies were not investigated.)

### Conclusions

In summary, on the basis of the present study, (i) CMAHKO in the pig may be critical to the success of clinical pig kidney xenotransplantation, and may be the most important after α1,3-galactosyltransferase gene be knockout, at least in Chinese patients; (ii) subjects with ESRD, or who are immunosuppressed after kidney allotransplantation, and brain-dead organ donors, all have lower levels of antibody binding and CDC to genetically-engineered pig cells than do healthy human volunteers; (iii) brain-dead subjects may mimic ESRD patients in that they both have low levels of anti-pig antibody levels, but experimental pig organ transplants in this group are unlikely to provide significant information of real value; (iv) TKO pigs with selected human ‘protective’ transgenes, e.g., CD55, are likely to prove to be optimal sources of kidneys for clinical xenotransplantation. (v) The role of SLAKO or SLA knockdown remains uncertain.

## Data Availability Statement

The raw data supporting the conclusions of this article will be made available by the authors, without undue reservation.

## Ethics Statement

The studies involving human participants were reviewed and approved by Ethics Committee of the Second Affiliated Hospital of Hainan Medical University. Written informed consent for participation was not required for this study in accordance with the national legislation and the institutional requirements. The animal study was reviewed and approved by ethics committee of the Hainan Medical University.

## Author Contributions

YW and GC designed the experiments. TL, HF, JD, QX, and SH participated in the performance of the research and data analysis. DP provided transgenic pigs for experiments. TL, DC, and HJ prepared the figures and wrote the article. DC, YW, and GC critically revised the article. All authors contributed to the article and approved the submitted version.

## Funding

This study was supported in part by the Major Scientific and Technological Project of Hainan province (ZDKJ2019009) and Hainan Provincial Natural Science Foundation of China (821QN413). The funder was not involved in the study design, collection, analysis, interpretation of data, the writing of this article or the decision to submit it for publication. Work on xenotransplantation at the Second Affiliated Hospital of Hainan Medical University, is supported in part by the Major Scientific and Technological Project of Hainan province (ZDKJ2019009) and Hainan Provincial Natural Science Foundation of China (821QN413).

## Conflict of Interest

Author JD is employed by Chengdu Clonorgan Biotechnology Co., LTD.

The remaining authors declare that the research was conducted in the absence of any commercial or financial relationships that could be construed as a potential conflict of interest.

## Publisher’s Note

All claims expressed in this article are solely those of the authors and do not necessarily represent those of their affiliated organizations, or those of the publisher, the editors and the reviewers. Any product that may be evaluated in this article, or claim that may be made by its manufacturer, is not guaranteed or endorsed by the publisher.

## Abbreviations

Allo-TCMR, allograft recipients who were currently experiencing T cell-mediated rejection; β4GalNT2KO, beta-1,4-N-acety1 galactosaminyltransferase 2-knockout; CMAHKO, cytidine monophosphate-N-acetylneuraminic acid hydroxylase-knockout; CDC, complement-dependent cytotoxicity; DBD, brain-dead donors; ESRD, end-stage renal disease; GTKO, α1,3-galactosyltransferase-knockout; HLA, human leukocyte antigens; hCD55, human complement-regulatory protein, CD55; OWMs, Old World monkeys; PBMCs, peripheral blood mononuclear cells; RBCs, red blood cells; SLA, swine leukocyte antigens; WT, wild-type.
