# Supplementary material for: Serum Antibody Binding and Cytotoxicity to Pig Cells in Chinese Subjects: Relevance to Clinical Renal Xenotransplantation
Source: Front Immunol. 2022 Mar 10;13:844632. doi: 10.3389/fimmu.2022.844632 (PMC8996717; doi:10.3389/fimmu.2022.844632)
Supplement: Supplementary file 1 [file DataSheet_1.pdf]

Supplemental Figure 1

TKO/hCD55 PBMCs

GTKO/CMAHKO PBMCs

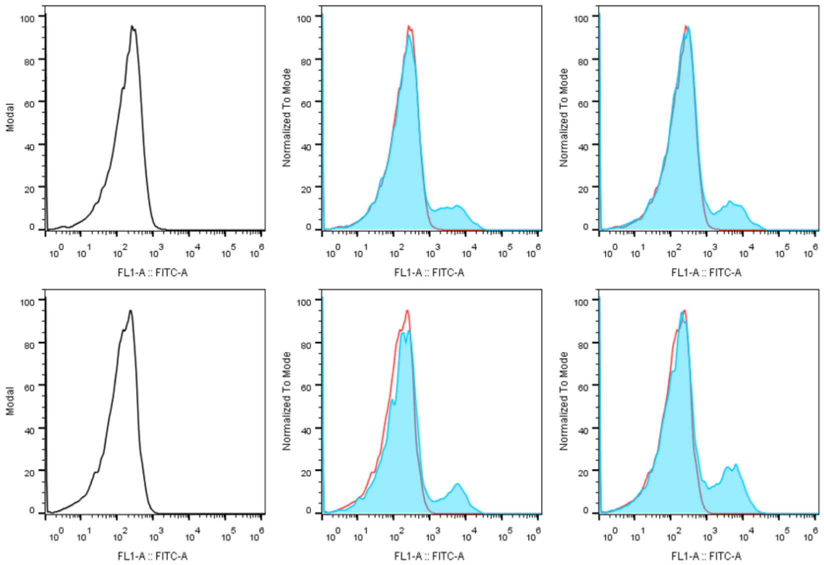

purified chicken anti-Neu5Gc antibody	-	-	+
Rabbit anti-Chicken IgY/ Alexa Fluor 555 antibody	-	+	+

Supplementary Figure 1: The false positive expression of Neu5Gc on pig PBMCs was caused by Rabbit anti-Chicken IgY/Alexa Flour 555 antibody. Blank histograms represent negative controls that were unstained cells **(Left)**. Pig PBMC was only stained with Rabbit anti-Chicken IgY/Alexa Flour 555 antibody **(Middle)**. Pig PBMC was stained with purified chicken anti-Neu5Gc antibody and then stained with Rabbit anti-Chicken IgY/Alexa Flour 555 antibody **(Right)**.
